# Multi-Factorial-Guided Media Optimization for Enhanced Biomass and Lipid Formation by the Oleaginous Yeast *Cutaneotrichosporon oleaginosus*

**DOI:** 10.3389/fbioe.2019.00054

**Published:** 2019-03-26

**Authors:** Dania Awad, Frank Bohnen, Norbert Mehlmer, Thomas Brueck

**Affiliations:** ^1^Werner Siemens-Lehrstuhl für Synthetische Biotechnologie, Technische Universität München, Garching, Germany; ^2^BBSI GmbH, Moosinning, Germany

**Keywords:** *Cutaneotrichosporon oleaginosus*, Response Surface Methodology, media optimization, FAMEs, biofuel, lipids

## Abstract

The non-conventional, oleaginous yeast *Cutaneotrichosporon oleaginosus* is flagged as an industrial cell factory for generation of oleochemicals and biofuels due to its substrate flexibility and high triglyceride yields. In this study, we employed a computational Response Surface Methodology to guide and streamline the experimental media optimization matrix with 12 nitrogen and 10 carbon sources in order to provide for high biomass and lipid accumulation toward an industrially relevant fermentation process. The resulting data provide new insights into *C. oleaginosus* physiology under variable nutritional states. Accordingly, the lipid content % (lipid weight/yeast dry weight) is controlled by a defined interplay between carbon and nitrogen. In our experimental setup, the highest biomass (18.4 ± 2.20 g/L) and lipid yield (9 ± 0.34 g/L; 49.74 ± 5.16% g lipid weight/g yeast dry cell weight) were obtained with lactose and yeast extract as carbon and nitrogen sources at an elemental weight ratio of 120:1, respectively. Interestingly, with ammonium salts as a N-source, the intracellularly accumulated triglycerides increasingly contain saturated fatty acids, which provides a new route to generate tailored fatty acid profiles for specific oleochemicals or food applications. Our data indicate that a metabolic ceiling for lipid accumulation in *C. oleaginosus* is obtained with the correct carbon and nitrogen source mixture.

## Introduction

Dwindling fossil resources and climate change drive the development of sustainable bio-based processes for generation of chemicals, pharmaceuticals, and biofuels, such as biodiesel (Chisti, 2007; Tai and Stephanopoulos, 2013). With regard to biofuel products, current biodiesel is derived from plant-oils, which are associated with limited renewability due to utilization of agricultural land mass and fresh water resources as well as application of chemical fertilizers and pesticides. Therefore, this first-generation biofuel merely offers a partial solution with respect to sustainability and drives the socioeconomic food vs. fuel debate, which has limited the industrial roll out of biofuel processes (Hill et al., 2006). In that regard, current arable lands are not sufficient to meet the global-demand for plant derived biofuels (Smith et al., 2010). Furthermore, rising demands for biodiesel lead to increasing prices of conventional plant oils. This situation lead to 2-fold price increase for rapeseed, peanut, and sunflower oils between 2007 and 2008 (Papanikolaou and Aggelis, 2011). Assuming a consistent pricing trend, 2020 price projection for the average vegetable oils are expected to exceed 2$.kg^−1^, which does not alleviate current economic or ecological pressures for biodiesel production (Koutinas et al., 2014). While algae based oils (3rd generation biodiesel) present an ecological alternative to plant oils, their photoautotrophic production is still associated with significant cost (5.6–21.0 $.kg^−1^) and technological barriers, including slow growth rate and difficulty in maintaining high-density cultures (Papanikolaou and Aggelis, 2011). Consequently, the development of new biofuel processes that utilize biomass waste without impacting agricultural activity are in demand to ensure global food security and address requirements for sustainable energy and oleochemical supply.

To that end, oleaginous yeasts offers a potentially sustainable alternative to meet the demand for bio-based energy and platform chemicals as they can fermentatively convert a broad spectrum of waste biomass substrates into triglycerides. Moreover, these organisms, can be cultivated in existing bioreactor systems without impacting agricultural activities (Li et al., 2008). Specifically, yeasts that intracellularly accumulate neutral lipids in excess of 20% w/cell dry weight when cultivated under nutrient (N, P limitation) restriction, are termed “oleaginous yeasts” (Ratledge and Wynn, 2002; Beopoulos et al., 2009). The majority of the generated triacylglycerols (TAGs) display a fatty acid profile similar to that of plant oils (Papanikolaou et al., 2013). Moreover, oleaginous yeasts, such as *Cutaneotrichosporon oleaginosus* (ATCC 20509) have a pronounced ability to utilize diverse carbon sources—such as glucose, galactose, xylose, n-acetylglucosamine, volatile fatty acids, cellobiose, sucrose, lactose, glycerol, and complex biomass waste materials as feedstock (Evans and Ratledge, 1983; Wu et al., 2010; Gujjari et al., 2011; Chi et al., 2012; Liang et al., 2014b; Willis et al., 2014; Liu et al., 2015; Rakicka et al., 2015). As this organism has the metabolic capacity to accumulate in excess of 50% w/cell dry weight as lipids when nutrient restriction is applied, it is in focus for industrial developments of sustainable oleochemical and biofuel production. Most interestingly, the fatty acid composition of *C. oleaginosus* is similar to that of plant-oils with C16 and C18 being the principal fatty acids (FAs) at 16–33 and 43–57% (g FAME/g dry yeast cell weight), respectively (Hassan et al., 1994a; Ageitos et al., 2011). In fact, B20 Biodiesel derived from *C. oleaginosus* microbial-oil meets the ASTM (D6751) certification (Wahlen et al., 2013; Willis et al., 2014). Being a suitable building block for biodiesel production, this microbial oil produced by oleaginous yeasts such as *C. oleaginosus* offers a sustainable alternative for plant-derived oil without competing with agricultural activities. Nonetheless, production cost of yeast SCO remains higher than that of 1st generation biofuel. While the former is estimated to 3 $.kg^−1^, excluding feedstock cost, the latter ranges between 1.2 and 1.9 $.kg^−1^ for conventional vegetable oils such as rapeseed, soybean, and sunflower oil (Papanikolaou and Aggelis, 2011). Techno-economic evaluation of SCO processes has demonstrated that the main cost generators are associated with the fermenters required to maintain fed-batch conditions (Koutinas et al., 2014; Karlsson et al., 2016).

To meet economic boundary conditions for industrial implementation of the technology, process costs have to be significantly reduced. In that regard, innovative fermenter designs, recycling/valorization of process residues streams and low energy oil recovery and purification methods are key to the commercialization of microbial oil production technology (Koutinas et al., 2014; Karlsson et al., 2016). In the same manner, the feedstock materials represent a considerable fraction of the overall fermentation-based production costs (Singh et al., 2016). To that end, several strategies could be implemented to significantly improve the techno-economic boundary conditions of microbial oil production. These include the utilization of various biogenic waste materials as low or negative cost feedstocks, including as raw glycerol (biodiesel residue stream) and lignocellulosic hydrolysates (Zhao et al., 2016). Co-fermentation of lignocellulosic-derived carbohydrates containing a mixture of pentoses (e.g., xylose) and hexoses (e.g., glucose) could also alleviate process costs (Stephanopoulos, 2007). Beyond the use of low cost hydrolysates, the design of zero waste yeast oil processes, that valorize all process streams lead to a significant economic and ecological enhancement in the overall process viability. In that regard, our group has recently reported a zero waste yeast oil production process based on marine macroalgae hydrolysates, where feedstock residues have been utilized as biological absorbents for recovery/recycling of industrially relevant metals (Ce^+3^, Cu^+2^, and Ni^+2^) from oil refining processes or for the removal of heavy metals (Pb^+2^) from waste water streams. Additionally, due to its unique sugar composition, the spent *C. oleaginosus* cell wall after oil extraction was proposed as a performance animal feed additive to improve the techno-economic viability of the overall process (Masri et al., 2018). Another strategy to increase the economic viability of the yeast oil process, involves genetically engineering oleaginous yeast strains to improve substrate uptake and direct the metabolic flux into lipogenic pathways, thereby improving lipid productivity and tailor process specific FA profiles (Mlickova et al., 2004). Despite the high cost of manufacture (COM), microbial production of specialty lipids can achieve higher market prices and therefore rapid market viability. To that end, production of cocoa butter substitutes (CBS) from oleaginous yeast strains has been demonstrated at a technical scale since 1980s. However, recent records anticipate generalized market penetration of CBS in food industry following indications of future disappearance of cocoa plant (Davies and Holdsworth, 1992; Smit et al., 1992; Hassan et al., 1994b; Papanikolaou and Aggelis, 2011). In a specific genetic engineering approach, a Δ9 fatty acid desaturase from *C. oleaginosus* has been cloned and characterized for the production of CBS-SCOs (Papanikolaou and Aggelis, 2011). Beyond the production of cocoa butter, genetic engineering approaches to generate high value cosmetic fats, such as shea butter and sal fat has been explored as cocoa butter equivalents (CBE) (Koutinas et al., 2014). Prior to technical implementation of any of these strategies, assessment and optimization of cultivation media components is essential to determine which factors influence biomass and lipid formation (Singh et al., 2016).

Identifying and designing optimal fermentation conditions plays a major role in the development of bioconversion systems, as the cultivation medium composition dictates product yield and volumetric productivity (Singh et al., 2016). In that respect, the carbon source is regarded as the most crucial element in the medium; as it represents the main energy source for growth, biomass formation, and subsequent lipid yield (Willis et al., 2014). In addition to the carbon source, the source of nitrogen is thought to be another essential factor determining formation of yeast biomass and lipids. In fact, selected nitrogen sources can elevate biomass productivity, while others may prevent the synthesis of certain metabolites (Singh et al., 2016). The biochemical basis of lipid accumulation in oleaginous yeasts is believed to be an adaptive response under a carbon-rich and nutrient-limited (mainly nitrogen) conditions, where excess carbon is incorporated into intracellular lipids (TAGs) as a form of energy storage (Willis et al., 2014). Furthermore, fatty acid composition differs greatly depending on the type and concentration of the carbon and nitrogen sources. Lipid yield and type, in addition to biomass formation, are likewise influenced by the carbon to nitrogen ratio (C: N) in addition to media components like carbon and nitrogen-sources as well as vitamins and minerals (Granger et al., 1993; Ratledge and Wynn, 2002). Enhancing strain genetics and improving media parameters are usually concomitant. *C. oleaginosus* ability to produce high amounts of intracellular lipids from a variety of substrates has previously been reported (Evans and Ratledge, 1983; Gujjari et al., 2011; Willis et al., 2014). However, genetically engineering this particular yeast remains a challenge. With many genes lacking functional annotations and absence of proteomic data, these engineering efforts only rely on recently reported transcriptomic sequences (Kourist et al., 2015). Recently methods that enable genetic accessibility of *C. oleaginosus* have been reported. These methods allowed an increase in total lipid yields as well as the generation of tailor-made, non-native fatty acid, such as polyunsaturated very long chain fatty acids eicosatrienoic and eicosadienoic acid and (E-10, Z-12) conjugated linoleic acid (Gorner et al., 2016). Based on the available data, this study will focus on a systematic, multi-factorial analysis of how carbon and nitrogen sources affect *C. oleaginosus* biomass and lipid formation.

To that end, conventional medium optimization studies are conducted following the classical one-factor-at-a-time (OFAT) method where, at a given time, only one factor is varied while all other variables remain constant. This strategy includes iterative removal, supplementation and replacement experiments of the various chemical and physical components of the medium, resulting in laborious protracted experiments (Singh et al., 2016). By contrast, design of experiment (DOE) guided studies, applied in this work, allow an accurate statistically validated, multi-factorial medium optimization strategy. This methodology reduces experimental time and labor compared to the OFAT method and generates statistically verified data. One approach to DOE is Response Surface Methodology (RSM), developed by Box and Wilson (Box and Wilson, 1951). RSM is a mathematical modeling algorithm that uses factorial designs to assess the correlation between the response and different variables either alone or in combination, eventually allowing the optimization of the production processes (Singh et al., 2016).

The aim of this study is to use RSM as a computational guidance tool to simultaneously modulate and optimize multiple medium factors, in particular carbon and nitrogen, to ultimately improve biomass and lipid production of the oleaginous yeast *C. oleaginosus*. Initially, the response surface methodology, applying a Box-Behnken Design, is employed to evaluate the effect of nitrogen and carbon levels. Specifically, RSM assess the relative contribution of carbon and nitrogen sources on the *C. oleaginosus* growth and lipid production. Subsequently, 12 distinct nitrogen sources and 10 selected carbon sources, encompassing the most comprehensive media components involving complex, synthetic/inorganic and organic media composition for *C. oleaginosus*, are investigated (Table 1). This statistically verified, multi-factorial data interrogation provides insight into the physiology of *C. oleaginosus* (growth, lipid production and fatty acid profile) toward development of an industrial cultivation process.

**Table 1:** Matrix of assayed media components.

**Compound number**	**Carbon source**	**Chemical nature**	**Nitrogen source**	**Chemical nature**
1	Glucose	Monosaccharide/Hexose	Ammonium chloride	Defined Inorganic
2	Galactose	Monosaccharide/Hexose	Ammonium sulfate	Defined Inorganic
3	Mannose	Monosaccharide/Hexose	Ammonium phosphate	Defined Inorganic
4	Fructose	Monosaccharide/Hexose	Calcium nitrate	Defined Inorganic
5	Sorbitol	Sugar alcohol	Potassium nitrate	Defined Inorganic
6	Xylose	Monosaccharide/Pentose	Sodium nitrate	Defined Inorganic
7	Arabinose	Monosaccharide/Pentose	Ammonium nitrate	Defined inorganic
8	Maltose	Disaccharide	Ammonium chloride + sodium nitrate	Defined Inorganic
9	Lactose	Disaccharide	Tryptone/Peptone derived Yeast Extract^*^	Complex Organic
10	Sucrose	Disaccharide	Yeast extract^*^	Complex Organic
11	–	–	Tryptone/Peptone^*^	Complex Organic
12	–	–	Urea	Defined Organic

* *Potential carbon and nitrogen sources*.

## Materials and Methods

### Yeast Strain and Inoculum Preparation

*Cutaneotrichosporon oleaginosus* ATCC 20509 (available in the laboratory culture collection of Werner Siemens Chair of Synthetic Biotechnology- WSSB, TU, Munich) was maintained on YPD (yeast extract peptone dextrose) agar plates (20 g/L peptone, 20 g/L agar, 20 g/L glucose, 10 g/L yeast extract). Stock cultures were transferred to fresh agar plates and stored at 4°C weekly. A single colony was transferred to 50 mL YPD liquid medium in 125 mL Erlenmeyer flask and cultured at 28°C for 24 h in a rotary incubator at 120 rpm prior to inoculation into prospective optimization media.

### Experimental Design

Media optimization can be divided into 3 stages, whereby the first stage assesses different carbon to nitrogen concentrations and ratios, the second and third stages compare the effect of different nitrogen and carbon sources on *C. oleaginosus* growth and lipid production, respectively. Aside from variation in carbon and nitrogen sources and concentrations, all cultures were comprised of 1.5 g/L MgSO_4_·7H_2_O, 0.4 g/L KH_2_PO_4_, 0.22 g/L CaCl_2_·2H_2_O in addition to trace elements: 1.2 mg/L (NH_4_)_2_SO_4_, 0.55 μ g/L ZnSO_4_·7H_2_O, 24.2 μg/L MnCl_2_·4H_2_O, 25 μg/L CuSO_4_·5H_2_O (adapted from Gorner et al., 2016). All shake flask experiments were carried out in 125 mL Erlenmeyer flasks with a total media volume of 50 mL. Identical culture conditions were maintained during optimization at 28°C in a rotary incubator at 120 rpm for 5 days. Three biological replicates were prepared from varied conditions listed below.

A total of 12 carbon to nitrogen ratios were prepared in a combinatorial system based on 8, 16, 24, and 36 g/L carbon and 0.13, 0.26, and 0.67 g/L nitrogen, with glucose and yeast extract as carbon and nitrogen sources, respectively. Elemental carbon to nitrogen weight ratios were covered in this study in the range of 12: 1 to 240: 1. Based on supplier's information (Carl Roth, Germany), the complex media—yeast extract and tryptone/peptone—contain 11.8% (w/w) and 10% (w/w) nitrogen, respectively. Following RSM analysis, various nitrogen and carbon sources were investigated using one-factor-at-a-time (OFAT) strategy. The various nitrogen sources evaluated in this study encompass: ammonium chloride, ammonium sulfate, ammonium phosphate, calcium nitrate, potassium nitrate, sodium nitrate, ammonium nitrate, 1:1 ammonium chloride and sodium nitrate, 1:1 yeast extract: tryptone/peptone, yeast extract, tryptone/peptone and urea. To investigate the effect of the nitrogen source on yeast growth, media was supplemented with 40 g/L glucose (16 g/L carbon) and nitrogen concentration of 0.13 g/L. The last stage of optimization covered 10 different carbon sources at an elemental carbon concentration of 16 g/L comprising 4 hexose monosaccharides (glucose, galactose, mannose, and fructose), a sugar alcohol (sorbitol), 2 pentose monosaccharides (xylose, arabinose), and 3 disaccharides (maltose, lactose, sucrose). For the screening of carbon sources, cultivation was carried out in media supplemented with the optimal nitrogen source as observed in the previous stage of optimization; 1.10 g/L yeast extract (0.13 g/L nitrogen).

### Analytical Methods

#### Gravimetric Method

Yeast growth was monitored daily by light scattering measurements at 600 nm. Dry Cell Weight (DCW) was quantified gravimetrically for 2 mL culture volume following harvesting, washing and lyophilization. Lipid extraction was conducted according to a modified procedure by Bligh and Dyer (1959) following yeast cells disruption using a high pressure homogenizer. Shortly, 6 mL of Folch's reagent (2:1 chloroform: methanol) was added to washed yeast cells. An incubation of 2 h was sufficient to transfer the cells' lipid content to the chloroform layer. The aspirated chloroform layer was later dried under nitrogen stream overnight and the extracted lipids were weighed.

#### Fatty Acid Profile Analysis

Fatty acid methyl esters (FAMEs) were obtained by methanol transesterification of lyophilized yeast biomass. The transesterification protocol was originally adopted from Griffiths et al. (2010) and modified in our lab by Gorner et al. (2016). FAME profiles were analyzed on a GC-2010 Plus gas chromatograph from Shimadzu (Nakagyo-ku, Kyoto, Japan) with flame ionization detector. One microliter sample was applied by AOC-20i auto injector (Shimadzu) onto a ZB-WAX column [30 m, 0.32 mm ID; 0.25 μm df; phenomenex (Torrance, CA, USA)]. The initial column temperature was set at 150°C (maintained for 1 min). A temperature gradient was applied from 150° to 240°C (5°C.min^−1^), followed by 6 min maintenance at 240°C. Fatty acids were identified according to retention times of the authentic standard: Marine Oil FAME Mix (Restek, USA). Individual FAME concentrations were based on peak areas relative to Methyl Non-adecaanoate C19 (Sigma, Germany), which was incorporated as an internal standard in all samples.

### Response Surface Methodology and Further Statistical Analysis

STATISTICA, version 7 (StatSoft Inc., Tulsa, USA) was adopted for design analysis of the first stage of media optimization. Analysis of Box-Behnken (response surface) design along with the analysis of variance (ANOVA) was used to estimate the appropriate statistical parameters. This design was selected for analyzing the effect of selected nutritional variables, namely carbon and nitrogen levels, present in growth media on the dependent variables: Biomass (g/L), Lipid weight (g/L) and Lipid content % (g lipid weight/ g dry yeast cell weight). The levels examined for each of the independent factors are displayed in Table 2. This statistical design involves interactions amongst the selected variables and follows a linear/quadratic approach for screening of factors. After discovering the significant factors, each of the dependent factors was individually fitted using a second-order polynomial equation and a multiple regression of the data was carried out for obtaining an empirical model to represent the most significant factors. The general form of the second-order polynomial equation is shown below:

$$\begin{array}{l} Y=\beta_{o}+\sum .\beta_{i}x_{i}+\sum \beta_{ii}x_{i}^{2}+\sum \beta_{ij}x_{i}x_{j}, \end{array}$$

**Table 2:** Box-Behnken Design of RSM for optimization of carbon and nitrogen concentration in cultivation media of *C. oleaginosus*.

**Run**	**C:N**	**X1**	**X2**	**Biomass**	**Lipid weight**	**Lipid content**	**Lipid yield**
		**(C (g. L^**−1**^))**	**(N (g. L^**−1**^))**	**(g. L^**−1**^)**	**(g. L^**−1**^)**	**(% g. g^**−1**^)**	**(g lipid. g^**−1**^ C)**
1	60	8	0.13	9.75 ± 1.09	3.25 ± 0.09	33.78 ± 4.68	0.04 ± 0.01
2	30	8	0.26	9.5 ± 0.11	1.82 ± 0.14	19.15 ± 1.53	0.23 ± 0.02
3	12	8	0.67	10.15 ± 0.13	1.39 ± 0.10	13.71 ± 1.10	0.17 ± 0.01
4	120	16	0.13	15.1 ± 0.60	6.69 ± 0.22	44.36 ± 2.56	0.42 ± 0.01
5	60	16	0.26	15.1 ± 0.41	3.18 ± 0.07	21.06 ± 0.48	0.20 ± 0.00
6	24	16	0.67	17.35 ± 1.10	2.33 ±0.29	13.48 ± 1.90	0.15 ± 0.02
7	180	24	0.13	16.85 ± 1.33	6.97 ± 0.76	41.66 ± 5.74	0.29 ± 0.03
8	90	24	0.26	19.7 ± 2.24	7.38 ± 1.01	38.09 ± 8.28	0.31 ± 0.04
9	36	24	0.67	23.95 ± 2.36	3.78 ± 0.61	15.98 ± 3.40	0.16 ± 0.02
10	240	32	0.13	15.4 ± 0.14	4.1 ± 0.47	26.64 ± 3.21	0.13 ± 0.01
11	120	32	0.26	22.9 ± 0.10	6.29 ± 0.73	27.48 ± 3.23	0.20 ± 0.02
12	48	32	0.67	24.3 ± 0.55	5.85 ± 0.14	24.09 ± 0.80	0.18 ± 0.00

*Elemental weight ratio of carbon to nitrogen were delivered by glucose and yeast extract, respectively*.

whereby, *Y* is the predicted response, β_*o*_is the interception coefficient, β_*i*_ is the linear coefficient, β_*ii*_ is the quadratic coefficient, β_*ij*_ is the interaction coefficient. The quality of the regression equations were judged based on the coefficient of determination *R*^2^ and Lack of fit *F*-test, given the availability of center points replicates, and possibility of pure error calculation. Optimal levels of carbon and nitrogen were inferred from analysis of response surface plots.

## Results and Discussion

### *In silico* Guided Optimization of Carbon and Nitrogen Concentrations Using Response Surface Methods

**Figure 1:**
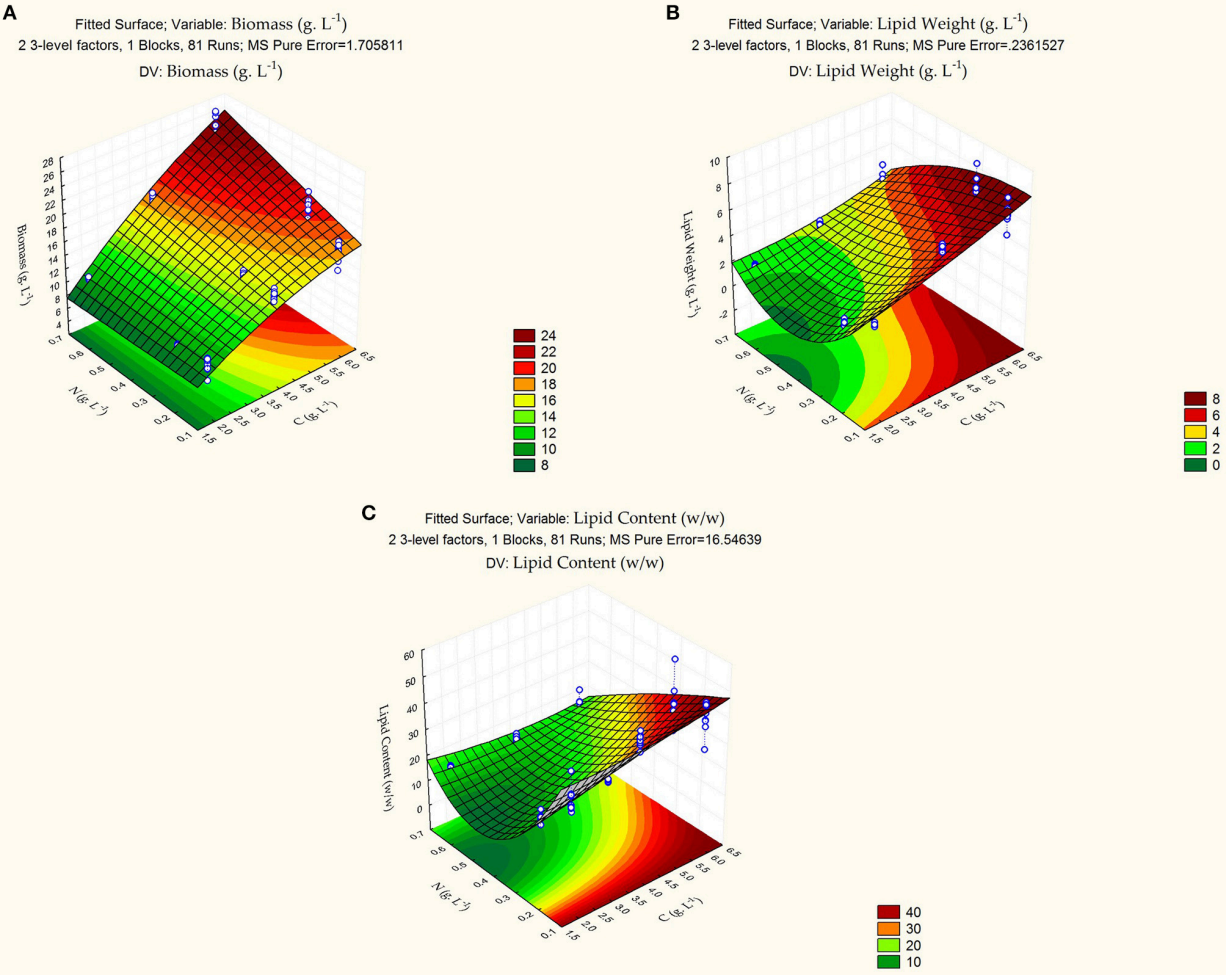
3D Response surface plot of the combined effects of carbon and nitrogen levels on **(A)** growth (g/L), **(B)** lipid weight (g/L), and **(C)** lipid content (g lipid weight/g dry yeast cell weight). Gradient legends display extent of measured factor. White points mark measured levels on which the Box-Behnken model was built.

To assess the effect of carbon and nitrogen concentrations in the cultivation media on growth and lipid production of *C. oleaginosus*, the composition was subjected to Box-Behnken design-based DOE analysis. This analysis was performed using glucose as carbon source, since yeasts are reported to harbor efficient glucose import systems. Similarly, yeast extract was used as a complex nitrogen source as it contains all essential amino acids as well as other nutritional factors such as fatty acids, vitamins and trace element, which in combination improve biomass and lipid yield [30, 31]. It was previously reported that an elemental carbon to nitrogen ratio of 12: 1 does not deliver a nitrogen-limited environment for *C. oleaginosus*, hence a range from 12: 1 to 240: 1 was analyzed in this study to cover the range between non-limiting and limiting cultivation conditions [32]. Initially, each concentration was carefully developed to fit one of three equally spaced values (−1, 0, and +1) to sufficiently generate a quadratic model. The model was later validated following an experimental set up of selected theoretical parameters. The Box-Behnken design matrix of the independent variables and their corresponding response on the dependent factors are displayed in Table 2. The fitted regression equations for each of the dependent factors are:

$$\begin{array}{lll} Y\left(\text{Biomass}\right) & = & -2.40+8.16\text{X1}-0.92\text{X12}+28.79\text{X2}\\ -32.40\text{X22}-25.06\text{X1X2}+28.26\text{X1X22}\\ +4.22\text{X12X2}-4.40\text{X12X22},\\ Y\left(\text{Lipid Weight}\right) & = & 7.11+0.13\text{X1}-42.45\text{X2}+50.45\text{X22}\\ +4.70\text{X1X2}-7.58\text{X1X22}+0.35\text{X12X2}\\ -0.32\text{X12X22},\\ Y\left(\text{Lipid Content}\right) & = & 69.71-2.76\text{X1}-297.026\text{X2}+328.79\text{X22}\\ +18.97\text{X1X2}-29.14\text{X1X22}+3.13\text{X12X2}\\ -3.64\text{X12X22}, \end{array}$$

whereby, X1 and X2 represent carbon and nitrogen levels, respectively. Table 3 summarizes the test for significance and adequacy of each of the regression models listed above. The optimal values for the investigated dependent factors were predicted from the 3D response surface plot. Figure 1 illustrates the effects of the independent variables and their combined effect on biomass (g/L), lipid weight (g/L), and Lipid content % (g lipid weight/ g yeast dry cell weight). Accordingly, the optimal predicted values for the dependent factors are 15.1 ± 0.60 g/L, 6.69 ± 0.22 g/L, and 44.36 ± 2.56% (g lipid weight/g dry yeast cell weight), respectively. The determination confidence coefficient (*R*^2^), which is conventionally applied to determine the model validity, implies that sample variation of 92.59, 95.64, and 90.23% for biomass, lipid weight, and lipid content, respectively, are attributed to the independent variables. Together with the significance of the Lack-of-fit test (*p*-values of 0.0928, 0.1085, and 0.1436), we determined the integrity of the model designed for this analysis (Table 3). As depicted in Table 2, the total growth increases with increasing carbon concentrations until a saturation level is obtained at 60 g/L glucose. Moreover, nitrogen limitation, which is essential for high lipid production, possess a strong negative influence on *C. oleaginosus* growth. For this reason, lipid content (g lipid weight/g dry yeast cell weight), as well as lipid weight (g/L), are equally scrutinized in this study. Furthermore, it is apparent from the three formulated regression equations that a combined effect of both independent factors significantly affects growth and lipid production. The second order polynomial equation was found to explain the optimal conditions by only considering the significant terms. Applying the RSM methodology allowed the role of carbon and nitrogen sources to be differentiated with respect to their importance and contribution to growth and lipogenesis potential. Those factors affecting Biomass are % C (linear and quadratic), % N (linear), and the linear interaction between % C and % N. In addition to those factors, the quadratic effect of % N highly influences lipid accumulation. Nitrogen-limitation has been previously assumed to be the strongest inducer of lipogenesis in *C. oleaginosus* [26]. The response surface plot (Figure 1) clearly depicts the influence and sensitivity of the respective factors on growth and lipid production. The currently incomplete comprehension of the metabolic network and regulatory mechanisms driving the lipogenic processes in *C. oleaginosus* might provide an explanation for our inability of attaining a higher determination coefficient [26]. Provided, that *C. oleaginosus* performs similarly at elemental carbon to nitrogen ratios of 120: 1 and 180: 1, further optimizations were carried out at C: N of 120:1 at concentrations of 16 g/L carbon and 0.13 g/L nitrogen, to reduce optimization process cost. The validity of the designed Box-Behnken model is evident in reproducible measurements of *C. oleaginosus* growth and lipid yields [33]. This particularly holds true when the organism is cultivated (C: N 120: 1, 16 g/L carbon and 0.13 g/L nitrogen) with optimal nitrogen (see Table 4, yeast extract) and carbon (see Table 5, glucose) ratios. In addition to relative amounts of carbon and nitrogen, different elemental concentrations at same carbon to nitrogen ratios were examined. While runs 1(8 g/L carbon and 0.13 g/L nitrogen) and 5 (16 g/L carbon and 0.26 g/L nitrogen) maintain a carbon to nitrogen elemental weight ratio of 60: 1, their variation in carbon and nitrogen concentrations results in distinct biomass, lipid weight and lipid content. Similar observations were recorded for runs 4 and 11 at C: N of 120: 1.

**Table 3:** Analysis of Variance (ANOVA).

**Source**	**Sum of squares**	**Degrees of freedom**	**Mean square**	***F*-value**	***p*-value**
Dependent Variable = Biomass (g. L^−1^); *R*^2^ = 0.92592; Adj. *R*^2^ = 0.92203; MS Pure Error = 1.705811					
X1 (L+Q)	1555.49	2.00	777.74	455.94	0.0000
X2	152.83	1.00	152.83	89.59	0.0000
X1X2	95.10	1.00	95.10	55.75	0.0000
Lack of Fit	14.17	4.00	3.54	2.08	0.0928
Pure Error	122.82	72.00	1.71	–	–
Total SS	1849.25	80.00	–	–	–
Dependent Variable = Lipid Weight (g. L^−1^); R^2^ = 0.95642; Adj. R^2^ = 0.95224; MS Pure Error = 0.2361527					
X1 (L)	184.11	1.00	184.11	779.60	0.0000
X2 (L+Q)	132.88	2.00	66.44	281.35	0.0000
X1X2	49.61	4.00	12.40	52.52	0.0000
Lack of Fit	0.62	1.00	0.62	2.64	0.1085
Pure Error	17.00	72.00	0.24	–	–
Total SS	404.42	80.00	–	–	–
Dependent Variable = Lipid Content (w/w); *R*^2^ = 0.90226; Adj. *R*^2^ = 0.89288; MS Pure Error = 16.54639					
X1	1098.95	1.00	1098.95	66.42	0.0000
X2 (L+Q)	8828.59	2.00	4414.30	266.78	0.0000
X1X2	1230.42	4.00	307.61	18.59	0.0000
Lack of Fit	36.18	1.00	36.18	2.19	0.1436
Pure Error	1191.34	72.00	16.55	–	–
Total SS	12558.62	80.00	–	–	–

**Table 4:** Results of cumulative growth and lipid production by *C. oleaginosus* cultured in parallel with variable nitrogen sources at elemental nitrogen concentration of 0.13 g. L^−1^,holding all other conditions identical [C: N of 120: 1 and 40 g. L^−1^ glucose (16 g. L^−1^ carbon)].

**Nitrogen source**	**Biomass (g. L^**−1**^)**	**Lipid weight (g. L^**−1**^)**	**%Lipid (g. g^**−1**^)**
Ammonium chloride	1.93 ± 0.08	0.32 ± 0.03	16.35 ± 1.78
Ammonium sulfate	2.17 ± 0.20	0.35 ± 0.04	16.37 ± 1.97
Ammonium phosphate	2.12 ± 0.10	0.51 ± 0.06	24.27 ± 3.39
Calcium nitrate	2.24 ± 0.17	0.73 ± 0.02	32.72 ± 2.89
Potassium nitrate	2.21 ± 0.12	0.91 ± 0.09	41.28 ± 5.64
Sodium nitrate	2.46 ± 0.13	1.06 ± 0.10	43.3 ± 4.18
Ammonium nitrate	1.73 ± 0.08	0.39 ± 0.03	22.74 ± 2.62
Ammonium chloride + sodium nitrate	1.67 ± 0.12	0.3 ± 0.03	18.09 ± 2.17
Yeast extract: Tryptone/Peptone	13.17 ± 0.27	6.37 ± 0.22	48.44 ± 2.30
Yeast extract	13.29 ± 0.15	7.1 ± 0.50	53.41 ± 4.12
Tryptone/Peptone	11.18 ± 0.18	5.58 ± 0.32	49.94 ± 3.64
Urea	4.96 ± 0.07	1.82 ± 0.13	36.75 ± 2.82

**Table 5:** Results of cumulative growth and lipid production by *C. oleaginosus* cultured in parallel with variable carbon sources at elemental carbon concentration of 16 g. L^−1^,holding all other conditions identical [C: N of 120 and 1.10 g. L^−1^ yeast extract (0.13 g. L^−1^ nitrogen)].

**Carbon source**	**Biomass (g. L^**−1**^)**	**Lipid weight (g. L^**−1**^)**	**%Lipid (g. g^**−1**^)**
Glucose	15.1 ± 0.60	6.62 ± 0.23	44.3 ± 2.53
Galactose	12.4 ± 1.22	4 ± 1.29	33.36 ± 11.98
Mannose	13.7 ± 1.82	7.03 ± 1.19	52.83 ± 13.82
Fructose	17 ± 0.77	7.25 ± 2.07	43.11 ± 11.63
Sorbitol	4.5 ± 0.45	0.6 ± 0.09	13.41 ± 2.52
Xylose	15.3 ± 0.43	5.63 ± 1.06	36.56 ± 6.57
Arabinose	8.7 ± 0.75	2.07 ± 0.50	23.91 ± 6.66
Maltose	14.5 ± 1.67	6.12 ± 1.11	41.38 ± 7.73
Lactose	18.4 ± 2.20	9 ± 0.34	49.74 ± 5.16
Sucrose	10.3 ± 1.14	2.79 ± 0.58	27.52 ± 6.60

### Optimization of Carbon and Nitrogen Concentrations

Conventionally, laboratory medium compositions applied in initial research activities differ substantially from the medium in which the final production strain is expected to perform on a technical scale (Hahn-Hagerdal et al., 2005). Specifically, strain development is typically carried out in chemically-defined media to allow for strain-selection, easier metabolism definition, batch-to-batch variation exclusion and growth factor interference elimination (Zhang and Greasham, 1999). Conversely, complex and semi-defined media favored in industrial settings allow for greater biomass and metabolite yields, while complying with economic constraints (Cocaign-Bousquet et al., 1995). Complex media are composed of undefined components and quantities, such as biogenic wastes and hydrolysates. The complex nitrogen sources examined in this study include yeast extract and tryptone/peptone and their mixture. The multiplicity of carbon and nitrogen sources of synthetic, organic, and complex media presented in this study stands as future reference for strain improvements throughout developmental and industrial stages. The significance of the nitrogen source on *C. oleaginosus* growth and lipid formation is often neglected, placing more weight on carbon source performance (Swoboda, 1922; Evans and Ratledge, 1984; Godard et al., 2007; Gutierrez et al., 2013). Whereas, previous studies have focused on *C. oleaginosus* ability for sugars co-utilization (Yu et al., 2014; Gong et al., 2016; Meo et al., 2017), this work is directed toward equal scrutiny and comparison of the effect of distinct carbon and nitrogen sources on *C. oleaginosus* growth and lipid production. This comparison will aide in making an informed decision on the choice of genetic manipulations, that have to be implemented, to optimize sugar transport systems and streamline metabolic flux to enable efficient *C. oleaginosus* growth and lipid formation. Similar strategies have been adopted for increasing lipid yield and expanding the substrate utilization of *Yarrowia lipolytica* (Ryu et al., 2016; Spagnuolo et al., 2018).

**Figure 2:**
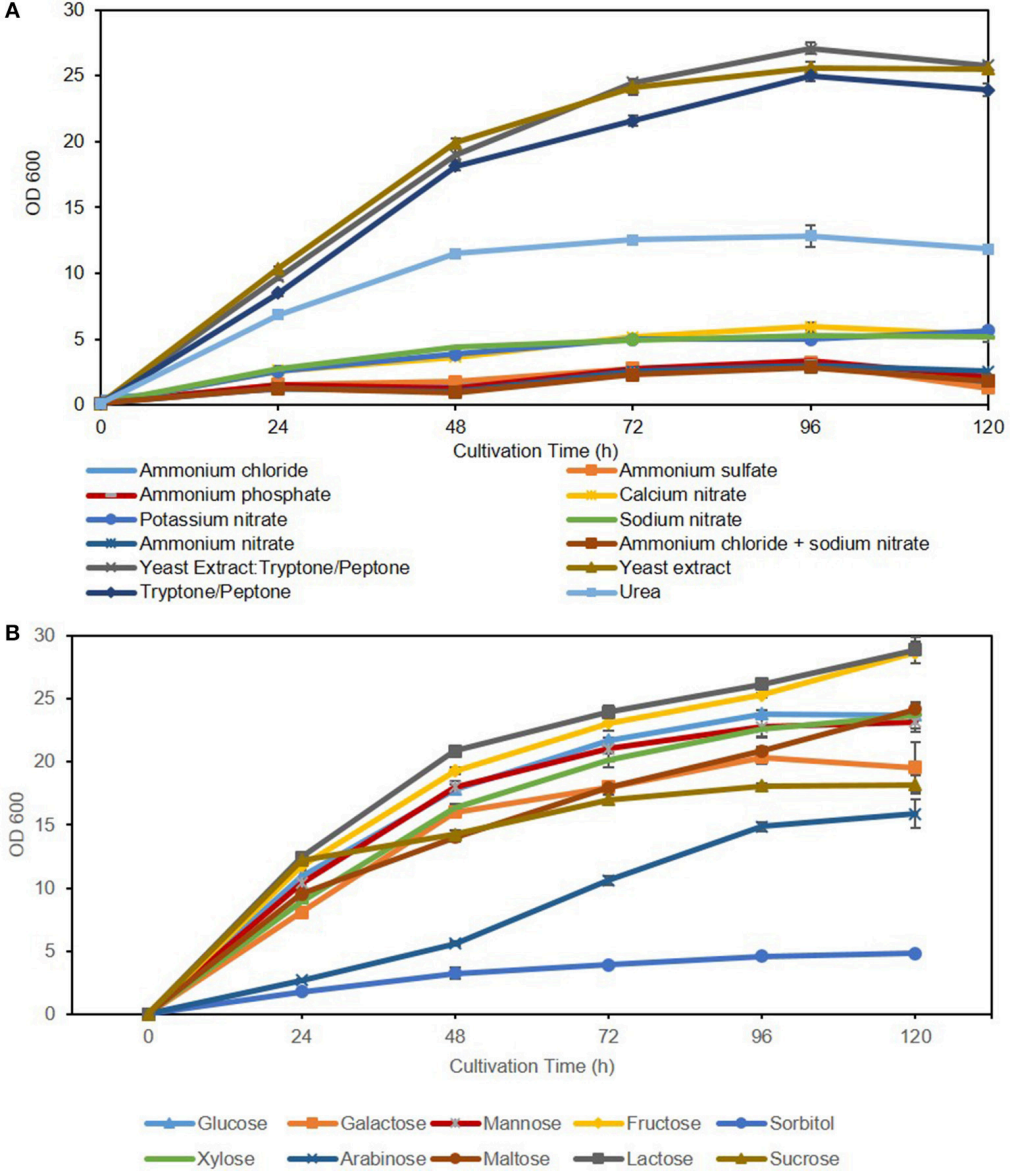
Effect of nitrogen source **(A)** and carbon source **(B)** on *C. oleaginosus* growth.

Commonly, media supplemented with complex (undefined/multiple) nitrogen sources result in high biomass accumulation due to ample availability of readily metabolizable amino acids, polypeptides, nucleotides, and various vitamins and trace elements (such as Mg, Ca, K, and Fe). This is apparent in Figure 2A and Table 4 with total biomass of 13.10 ± 0.27 g/L for yeast extract: tryptone/peptone, 13.29 ± 0.15 g/L for yeast extract and 11.18 ± 0.18 g/L for tryptone/peptone, after 120 h of cultivation. In contrast, low yeast biomass accumulation in media supplemented with synthetic/ inorganic nitrogen is attributed primarily to additional energy requirement for amino acid synthesis aside from energy requirement for self-propagation and growth. This is evident in media containing ammonium salts resulting in severely reduced growth compared to media containing complex nitrogen sources, followed by nitrates, with an average biomass of 2.1 ± 0.3 g/L. Additionally, ammonium uptake and growth is largely deterred by the acidification of the media in which excess protons, generated from amino acid biosynthetic pathway, are transported out of the cell to stabilize internal pH (Casey et al., 1983; Casey and Ingledew, 1986). Nonetheless, minimal potassium requirements for optimal yeast growth must be 100-fold higher than environmental concentrations (abundance of amino acids) when ammonium is used as the nitrogen source (Rodriguez-Navarro and Ramos, 1984; Hess et al., 2006). When synthetic nitrogen sources were utilized in this study, potassium levels were not adjusted accordingly, which could explain lower biomass measurements. Moreover, the target of rapamycin complex (TORC) associated signaling cascade is a key player in nutrient-starvation, which in oleaginous yeasts is linked to lipid accumulation and cell proliferation. In the model yeast *S. cerevisiae*, TORC is also linked to ammonium toxicity and chronological life span (CLS) shortening in absence of amino acids (Santos et al., 2013; Bracharz et al., 2017b). This correlation is yet to be established in oleaginous yeasts.

Our data indicates, that with respect to the lipid biosynthesis efficiency corrected for biomass yield, i.e., the lipid content % (g lipid weight/g dry yeast cell weight) is quantitatively comparable over various cultivation conditions. Conversely, the lipid weight (g/L) is directly proportional and influenced by the growth rate. With respect to the former, the lipid content of *C. oleaginosus* grown in nitrates surpasses those grown in ammonium salts. In that regard, a difference of 7.9% (g lipid weight/g dry yeast cell weight) in lipid content was found between *C. oleaginosus* cultivated in media supplemented with ammonium phosphate compared to equivalent cultivations with ammonium sulfate and ammonium chloride, elevated for the former. Additionally, no altered lipid formation response was detected in cultivation media containing different ammonium salts or different nitrates. Notably, coupling of ammonium salts with nitrate by supplementing media with ammonium nitrate or 1:1 ammonium chloride: sodium nitrate resulted in a similar lipid content as yeast grown in ammonium salts alone. This data is an indication that *C. oleaginosus* may be sensitive to ammonium toxicity.

Organic nitrogen was also supplemented to cultivation media in the form of urea. In this cultivation scenario, an intermediate growth between synthetic (ammonium salts and nitrates) and complex media (yeast extract and tryptone/peptone), resulting in an average biomass of 5.0 ± 0.1 g/L and similar lipid content as yeast grown in nitrate [36.8 ± 2.8% (g lipid weight/g dry yeast cell weight)] was recorded. However, the final lipid weight collected after 120 h of cultivation was higher for *C. oleaginosus* grown in urea compared to synthetic nitrogen sources due to enhanced growth. Initial isolation of *C. oleaginosus* from Iowa State University Dairy Farm and its ability to metabolize urea triggered the annotation of its urate catabolic pathway (Bracharz et al., 2017a). Such studies have shown that nitrogen limitation results in upregulation of urea transporter gene DUR3, urate oxidase gene URO1, putative ammonia transporter genes and most allantoin permeases to facilitate the import of remaining nitrogen sources (Kourist et al., 2015). Whereas, glucose delivers 16 g/L of carbon concentration into cultivation media, urea merely delivers 0.055 g/L, indicating that the contribution of urea to the carbon concentration of the cultivation media is insignificant in comparison with glucose. In analogy, the contribution of yeast extract to the carbon concentration (0.44 g/L) of the cultivation is insignificant in comparison to the various sugars examined at 16 g/L, especially at the minute amount of yeast extract used to maintain nitrogen-limited conditions.

Growth and lipogenic potentials of *C. oleaginosus* on variable carbon sources are depicted in Figure 2B and Table 5. In addition to its high lipogenic potential, the relatively high growth rates observed for this strain on a wide variety of sugar sources makes it highly desirable for industrial purposes, as fermentation costs can be reduced with the use of complex waste materials and hydrolysates. In contrast, the model oleaginous yeast *Y. lipolytica*, which has been target of extensive metabolic engineering efforts can only grow on few select carbon sources (glucose, mannose and glycerol) (Sitepu et al., 2014; Shi and Zhao, 2017). Assimilation of pentose sugars, mainly xylose, has been heavily investigated over the last 30 years for its abundance in lignocellulosic (30%) and hemicellulosic (90%) biomasses, which in turn are cost effective and highly abundant in nature (Tanimura et al., 2018). Recent efforts enabling co-metabolism of hexose and pentose sugars by overcoming glucose repression in various oleaginous yeasts including *Rhodosporidium toruloides* and *Rhodotorula glutinis* has been reported (Yamada et al., 2017). Adversely, few oleaginous yeasts including *C. oleaginosus, L. starkeyi*, and *Geotrichum fermentans* exhibit the natural ability for sugar co-metabolism, which makes them directly applicable for growth on complex fermentation substrates (Tanimura et al., 2018). Furthermore, semi-defined media can be selected more carefully with the aid of the results presented in this study. With respect to biomass yield and lipid formation, it can be concluded that *C. oleaginosus* performs well on all tested carbon sources except the sugar alcohol, sorbitol. To this end, no record of *C. oleaginosus* efficient growth on sugar alcohols was evident in literature. Adaptation and remodeling of sugar transport system to accommodate for arabinose appears slower in *C. oleaginosus* compared to other sugar transporters (Brauer et al., 2005). This is evident in the slow initial growth of this yeast on arabinose following pre-culturing on favorable sugar—glucose. In contrast, the galactose-glucose disaccharide lactose recorded highest cumulative growth and subsequently ranked amongst the highest with respect to lipid production efficiency (lipid content, g lipid weight/g dry yeast cell weight). Interestingly, lactose serves as a superior carbon source in *C. oleaginosus* fermentations compared to either of its monomers galactose or glucose. As a result of the high growth rate of *C. oleaginosus* when cultured on lactose, two lactose hydrolases have been identified and thoroughly studied (West et al., 1990). Given that *C. oleaginosus* was originally isolated from dairy farm, it may be particularly adapted for efficient lactose metabolism (Bracharz et al., 2017a). Conversion of whey permeate to lipids by this strain is industrially favorable given the high cell density and lipid yield achieved due to high lactose content (Bracharz et al., 2017a). *C. oleaginosus*-based lipid production in pilot scale of 500 L bioreactors using lactose-rich deproteinised cheese whey has been reported in New Zealand since 1988 (Davies, 1988). Interestingly, lipid content (49.7% g/g) achieved in this study during lactose fermentation is consistent with recently reported lipid content (49.6% g/g) recorded for *C. oleaginosus* grown on lactose-rich deproteinised cheese whey and wine lees hydrolysate (Kopsahelis et al., 2018). However, fed-batch cultivation conditions employed by Kopsahelis et al. allowed for high lipid titers of 33.1 g/L, compared to 9 ± 0.34 g/L achieved in shake-flask fermentation in this study. Similarly, spent coffee hydrolysate offers another cost-efficient feedstock for *C. oleaginosus*, due to its high mannose content (Table 5) (Scully et al., 2016; Orrego et al., 2018). With respect to the assayed sugar monomers, galactose performs intermediately between glucose (C-4 epimer) and mannose (C-2 epimer). Sugar co-utilization studies have previously demonstrated a delayed uptake for galactose, which may be due to less efficient transport systems across the cell membrane compared to glucose (Meo et al., 2017). The ketonic hexose, fructose, was shown to enhance the total growth of *C. oleaginosus* by a minimum of 1.9 g/L in comparison to other hexoses, nevertheless its lipogenic potential was not enhanced. Whereas, growth of *C. oleaginosus* on media containing fructose as sole carbon source was not previously reported, Tchakouteu et al. described initial abundance of intracellular total sugars (ITS) composed of glucose and fructose upon growth on sucrose followed by 66% drop concurrent with lipid production (Tchakouteu et al., 2015). At variance with either fructose or glucose, sucrose resulted in much lower biomass (10.3 ± 1.14 g/L) and lipid yields (2.79 ± 0.58 g/L; 27.52 ± 6.60% g lipid weight/g dry yeast cell weight). We further confirm reported work by Bracharz et al. that arabinose cannot be metabolized by *C. oleaginosus* to generate biomass. However, most interestingly its aldopentose diastereomer, xylose, is a more favorable substrate for biomass generation than glucose under the evaluated growth conditions (Liang et al., 2014a; Bracharz et al., 2017a). In addition, maltose, a disaccharide of glucose, mimicked glucose in both biomass and lipid weight, indicating that *C. oleaginosus* may harbor a very efficient extracellular maltose hydrolase activity or a very efficient maltose transporter in addition to an intracellular glycosidase shared with central metabolism, both of which have not been described to date.

### Effect of Nitrogen and Carbon Source on Fames Profile

**Figure 3:**
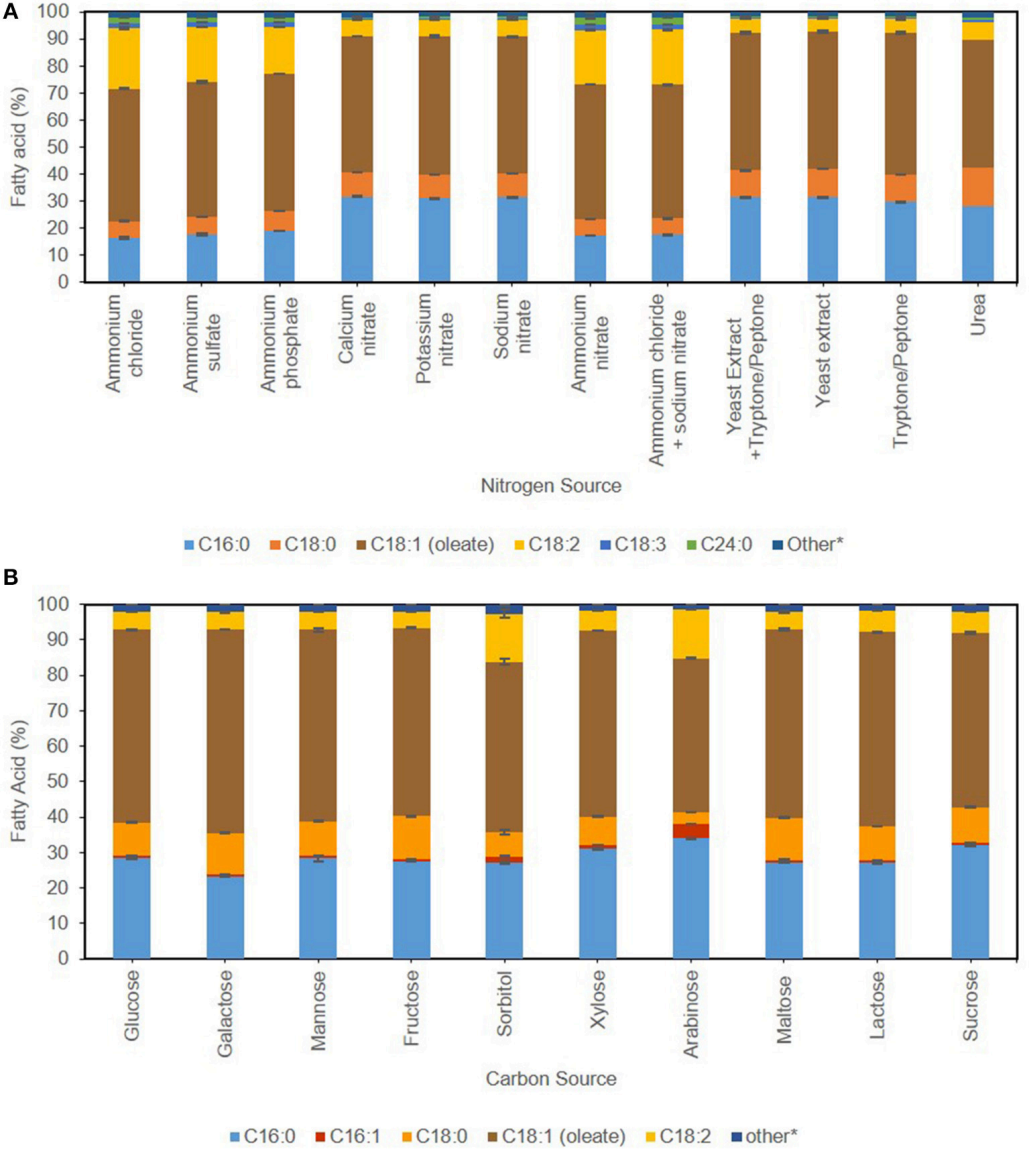
Effect of nitrogen source **(A)** and carbon source **(B)** on FAMES profile of *C. oleaginosus*. Other constitute fatty acids present below 1% of total profile, including C12:0, C14:0, C18:3, C20:0, C20:1, C20:3, C22:0, C24:0 for **(A)** and C12:0, C14:0, C18:3, C20:0, C20:1, C20:3, C22:0, C24:0 for **(B)**.

Industrial application of Single Cell Oils (SCO) is dependent on the fatty acid profile of the yeast in question, which in turn is influenced by cultivation conditions (Ochsenreither et al., 2016). Regardless of growth regime, the principal chemical composition of *C. oleaginosus* TAG mimics plant-derived lipids such as palm oil, with oleic acid [C18:1, 43–57% (g FAME/g dry yeast cell weight)] as main component succeeded by palmitic acid [C16:0, 16–33% (g FAME/g dry yeast cell weight)] (Hassan et al., 1994a). Increased concentrations of unsaturated fatty acids, as detected in *Rhodosporidium azoricum*, are essentially required to improve the cold-flowing properties of biodiesel (FAMEs). However, they are undesirable for Green-diesel production due to elevated hydrogen consumption. The maximal potential of *C. oleaginosus* is thus achieved in Green-diesel (HVO) processes, as it meets advanced feedstock specifications required by hydrotreatment process (Hydrogenated Vegetable Oil—HVO) due to its low degree of unsaturation (Capusoni et al., 2017). To achieve this level of saturation for medium chain fatty acids (MCFA), a recent study combines molecular design and TALENs in *Y. lipolytica* for the development of sustainable aviation fuels (Rigouin et al., 2017). The main influence exerted by media composition is most apparent in saturation degree perceived in Figures 3A,B. This is evident when ammonia is supplemented in the media, with a shift in the FA spectrum from palmitic acid [C16:0, 13% (g FAME/g dry yeast cell weight)] and stearic acid [C18:0, 4% (g FAME/g dry yeast cell weight)] toward linoleic acid [C18:2,15% (g FAME/g dry yeast cell weight)], in addition to assumed elongation with emergence of 1.8% (g FAME/g dry yeast cell weight) lignoceric acid C24:0. The fatty acid profile of *C. oleaginosus* appears to be more susceptible to carbon source than nitrogen source, as visualized in Figure 3B. Whereas mannose, maltose, and lactose attain identical fatty acid profile as glucose, sorbitol and arabinose show maximal diversion. The latter show reduction in stearic [C18:0, 2–6% (g FAME/g dry yeast cell weight)] and oleic acid [C18:1, 6–8% (g FAME/g dry yeast cell weight)] in favor of linoleic acid [C18:2, 8% (g FAME/g dry yeast cell weight)] in comparison with glucose. Additionally, arabinose along with xylose and sucrose, show a slight decrease in oleic acid C18:1 in favor of palmitic acid [C16:0, 3% (g FAME/g dry yeast cell weight)]. When galactose is used as a carbon source, the result involves a decrease in palmitic acid (C16:0) consistent with parallel increase in stearic acid (C18:0) and recording highest fraction of oleic acid (C18:1) at 57% (g FAME/g dry yeast cell weight). The differentiation among carbon and nitrogen sources is more prominent with the fatty acid profiles. Specific fatty acid profiles associated with desired product application can be obtained with careful selection of media components.

## Conclusion

Response Surface Methodology (Box-Behnken) allowed concurrent optimization of carbon and nitrogen concentrations and elemental ratios in cultivation media of *C. oleaginosus*, achieving a lipid yield of 0.42 ± 0.01 g lipid/g carbon, with 120:1 (16 g/L carbon and 0.13 g/L nitrogen). In our experimental setup, the highest biomass (18.4 ± 2.20 g/L) and lipid yield [9 ± 0.34 g/L; 49.74 ± 5.16% (g lipid weight/g dry yeast cell weight)] were obtained when lactose and yeast extract were used as carbon and nitrogen sources at an elemental weight ratio of 120:1, respectively. Conversely, the lowest biomass (1.93 ± 0.08 g/L) and lipid yield [0.32 ± 0.03 g/L, 16.35 ± 1.78% (g lipid weight/g dry yeast cell weight)] were obtained for glucose and ammonium chloride as carbon and nitrogen sources at an elemental weight ratio if 120:1, respectively. The physiological implications of these results are explained by the adaptive characteristics of *C. oleaginosus* to uptake and metabolize lactose and amino acids efficiently, in contrast to inorganic nitrogen entities. Furthermore, complex organic nitrogen sources such as yeast extract contain ample vitamins and trace minerals required for growth. Notably, the lowest lipid content [13.48 ± 1.90% (g lipid weight/g dry yeast cell weight)] was obtained with a C: N of 24:1 using glucose and yeast extract as a carbon and nitrogen sources, indicating that nutritional starvation was not reached. That the lowest biomass and lipid yields were obtained with ammonium chloride due to toxicity effects exerted by ammonium upon *C. oleaginosus* metabolism (see section Optimization of Carbon and Nitrogen Concentrations). These observations, in conjunction with the repertoire of this yeast's behavior in various carbon and nitrogen sources tested here, present a valuable source for identifying adequate and cost-efficient growth media (hydrolysates and waste materials). Comparison of fatty acid profiles of *C. oleaginosus*, when grown on different carbon and nitrogen sources, revealed enhanced saturation concerted with inorganic nitrogen sources, sorbitol and arabinose as carbon sources. Furthermore, it is pivotal to develop appropriate techniques for genetic manipulation to further improve the lipogenic potential and broaden the industrial application of *C. oleaginosus*. Future studies aimed at understanding *de novo* lipogenesis in *C. oleaginosus* from proteomic perspective will depend on optimal culture conditions developed in this study.

## Data Availability

All datasets generated for this study are included in the manuscript and/or the supplementary files.

## Author Contributions

Conceptualization of the study was conducted jointly by DA, NM, and TB. The methodological approach was designed and carried out by DA, FB, and NM. Data validation was jointly carried out by all authors. DA prepared the original draft of the manuscript. The manuscript was jointly finalized by all authors.

### Conflict of Interest Statement

FB was employed by company BBSI GmbH. The remaining authors declare that the research was conducted in the absence of any commercial or financial relationships that could be construed as a potential conflict of interest.
